# Population-level health impact of hypothetical waning 1-dose human papillomavirus vaccination and 2-dose mitigation strategies in a high cervical cancer burden setting

**DOI:** 10.1093/jncimonographs/lgae039

**Published:** 2024-11-12

**Authors:** Emily A Burger, Jean-François Laprise, Allison Portnoy, Jennifer C Spencer, Stephen Sy, Mary Caroline Regan, Élodie Bénard, Mélanie Drolet, Marc Brisson, Jane J Kim

**Affiliations:** Center for Health Decision Science, Department of Health Policy and Management, Harvard T.H. Chan School of Public Health, Boston, MA, USA; Department of Health Management and Health Economics, University of Oslo, Oslo, Norway; Centre de Recherche du CHU de Québec, Axe Santé des populations et pratiques optimales en santé, Université Laval, Québec, Canada; Center for Health Decision Science, Department of Health Policy and Management, Harvard T.H. Chan School of Public Health, Boston, MA, USA; Department of Global Health, Boston University School of Public Health, Boston, MA, USA; Department of Population Health, Department of Internal Medicine, University of Texas at Austin, Dell Medical School, Austin, TX, USA; Center for Health Decision Science, Department of Health Policy and Management, Harvard T.H. Chan School of Public Health, Boston, MA, USA; Center for Health Decision Science, Department of Health Policy and Management, Harvard T.H. Chan School of Public Health, Boston, MA, USA; Centre de Recherche du CHU de Québec, Axe Santé des populations et pratiques optimales en santé, Université Laval, Québec, Canada; Département de Médecine Sociale et Préventive, Université Laval, Québec, Canada; Centre de Recherche du CHU de Québec, Axe Santé des populations et pratiques optimales en santé, Université Laval, Québec, Canada; Centre de Recherche du CHU de Québec, Axe Santé des populations et pratiques optimales en santé, Université Laval, Québec, Canada; Département de Médecine Sociale et Préventive, Université Laval, Québec, Canada; Department of Infectious Disease Epidemiology, Imperial College, London, UK; Center for Health Decision Science, Department of Health Policy and Management, Harvard T.H. Chan School of Public Health, Boston, MA, USA

## Abstract

**Background:**

We simulated the impact of hypothetical waning scenarios of a 1-dose human papillomavirus (HPV) vaccination paired with switching to 2-dose mitigation strategies guided by empirical vaccine trial reporting timelines.

**Methods:**

Using 2 independent mathematical models fitted to a high-burden setting, we projected the cumulative cervical cancer cases averted over 85 years for alternative HPV vaccination scenarios under 2 program adoption timelines: 1) de novo introduction of a 1-dose HPV vaccination and 2) a switch from an existing 2-dose HPV vaccination program to a 1-dose vaccination. We assumed 80% vaccination coverage with the bivalent vaccine and an average duration of a 1-dose HPV vaccine protection of either 30 or 25 years with 100% efficacy. We varied the eligible age group(s) at program introduction and the 2-dose mitigation (single-age cohort or multi-age cohort). If needed for mitigation, reintroduction of 2-dose vaccination was assumed to occur in 2036 (ie, 30 years after initiation of the Costa Rica Vaccine Trial).

**Results:**

Under both vaccine adoption timelines, the models projected that countries could achieve the same level of health benefits by switching to 2 doses in 2036 using a multi-age cohort approach as with initiating a 2-dose or 1-dose vaccination program with no waning. With only a single-age cohort 2-dose mitigation approach, 98%-99% of cases would be prevented compared with the health benefits of 2 doses or a noninferior, durable 1 dose.

**Conclusions:**

Countries hesitant to adopt a 1-dose HPV vaccination program may have opportunities to leverage the benefits and efficiency of a 1-dose schedule while awaiting longer-term reporting from 1-dose durability studies, including Costa Rica Vaccine Trial.

Cervical cancer, primarily caused by persistent infection with a high-risk human papillomavirus (HPV), is preventable through prophylactic HPV vaccination (1,2). HPV vaccination is necessary to achieve the World Health Organization’s (WHO) goal of eliminating cervical cancer as a public health problem (3). Initiation of HPV vaccination programs has increased since the Strategic Advisory Group of Experts on Immunization (SAGE) made a permissive recommendation for a 1-dose HPV vaccination schedule in 2022 (4). SAGE concluded that a 1-dose HPV vaccination schedule has the potential to “accelerate introductions, reduce operational costs and complexity, and improve coverage” (4). Barriers to HPV vaccination program initiation may be reduced by a 1-dose rather than 2-dose schedule for preadolescents, considering the potential efficiency gains (ie, using half the number of doses required to prevent the same number of cervical cancers). However, many countries with the highest burdens of cervical cancer have not yet initiated any HPV vaccination program. By February 2024, the WHO’s HPV Vaccine Dashboard reported that a total of 143 countries worldwide have introduced HPV vaccination, and 37 countries recommend a 1-dose program (5).

Countries may be reluctant to initiate or switch to a 1-dose program despite SAGE and Regional Technical Advisory Groups on Immunizations recommendations as a 1-dose vaccination is not on the package insert and thereby considered an off-label use. Concerns regarding the durability of a 1-dose vaccine schedule, and to a lesser extent a 1-dose efficacy [given the growing evidence of noninferiority (6)], may contribute to continued hesitancy to either initiate a 1-dose HPV vaccination program or switch the preadolescent routine program from a 2-dose to a 1-dose schedule. Despite potential durability concerns, countries may have opportunities to leverage the benefits and efficiency gains of a 1-dose schedule while awaiting the long-term reporting from 1-dose durability studies, such as the Costa Rica Vaccine Trial (CVT) Long Term Follow-Up Study (7) or the International Agency for Research on Cancer Indian cohort study (8). The CVT, which started in 2005 and has thus far shown up to 16 years of sustained antibody levels from a 1-dose vaccination, would be expected to be among the first trials to detect potential waning after 25 or 30 years (in the years 2031 and 2036, respectively) (9).

Given the natural history of HPV infection progression to cervical cancer, understanding the long-term impact of initiating or switching to a 1-dose HPV vaccination program under hypothetical waning scenarios requires the use of mathematical simulation models. In addition, the resilience of HPV vaccination programs to mitigation approaches that minimize potential losses in health benefit (ie, compared with 2-dose vaccination) may depend on when the program was introduced, the number of age cohorts vaccinated, and the coverage level of the programs.

Previous modeling studies have projected that a 1-dose vaccination could allow for extension of vaccination programs to boys or older women to maximize the population-level effectiveness in low- and middle-income countries (LMICs) (10). Modeling studies have also estimated how immediate implementation of a 1-dose program prior to confirmatory noninferiority studies can expedite health benefits (11). However, no comparative modeling studies have assessed hypothetical 2-dose mitigation strategies (ie, switching from a 1-dose to a 2-dose program) that are paired with the reporting timeline of empirical 1-dose vaccine durability studies, such as CVT. Given these alternative HPV vaccine implementation timelines and target populations, understanding the long-term health effects under potential 1-dose HPV vaccine waning scenarios may guide decision makers to understand a realistic timeline for switching to a 2-dose mitigation strategy (if needed) before health impacts on the population are realized. Using a comparative model-based approach in the context of a setting with high cervical cancer burden, we aimed to identify the impact of different hypothetical waning scenarios for 1-dose HPV programs paired with reverting to a 2-dose program that is guided by empirical vaccine trial reporting timelines.

## Methods

### Analytic overview and scenarios

We used 2 independent simulation models (Harvard [Harvard T.H. Chan School of Public Health] and HPV-ADVISE [Université Laval]) that reflect HPV transmission dynamics to capture the impact of HPV vaccine-related dosing decisions on cervical cancer over time, including herd effects. Both models were fitted to a high-burden setting (age-standardized cervical cancer incidence rate >50 per 100 000 women) to project age-standardized cervical cancer incidence rates over an 85-year period under alternative HPV vaccination program characteristics.

**Figure 1.**
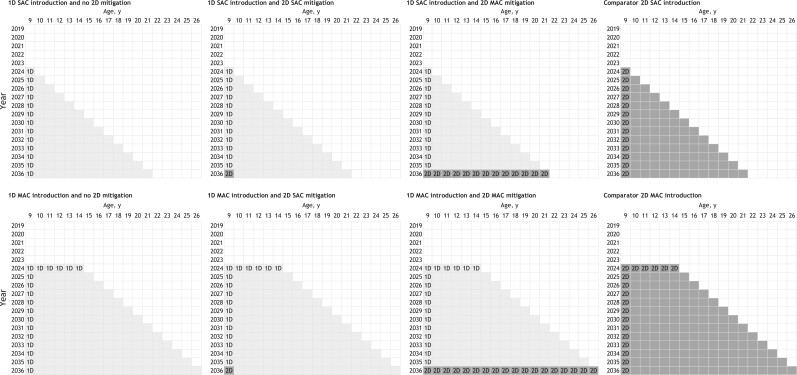
Primary analysis scenarios contextualized for new adopter countries that consider de novo introduction of a 1-dose human papillomavirus (HPV) vaccination program in 2024 assuming a 1-dose HPV vaccine would provide an average of 30 years of protection prompting a switch to a 2-dose program in 2036 (ie, the 30-year reporting timeline for the Costa Rica Vaccine Trial). Program introduction and 2-dose mitigation vary by the eligible age group(s), that is, single-age cohort age 9 years (y) or additionally providing multi-age cohort vaccination approach. One-dose scenarios ranged from 1-dose single-age cohort introduction in year 2024 with no 2-dose mitigation (**top left panel**) to 1-dose multi-age cohort introduction (ages 9-14 years) in 2024 with a switch to a 2-dose multi-age cohort mitigation in 2036 (age 9-26 years) (**bottom row, panel second from the right**). 1D = 1-dose; 2D = 2-dose; MAC = multi-age cohort; SAC = single-age cohort.

To isolate the potential impact of hypothetical 1-dose vaccine waning and compare mitigation scenarios for countries that have different HPV vaccination program adoption timelines, our primary analysis was contextualized for countries considering de novo introduction of a 1-dose HPV vaccination program in 2024 (new adopters) (see Figure 1). In contrast, our secondary analysis was contextualized for countries with a prior 2-dose HPV program introduction in 2019 [based on the introduction timeline for several high-burden countries (5)], followed by a hypothetical switch to a 1-dose schedule 5 years later in 2024 (switchers) (Supplementary Figure S1, available online).

For the primary and secondary analyses, we varied the vaccine eligible age group(s) at program introduction and 2-dose mitigation (ie, single-age cohort or inclusion of a 1-year multi-age cohort catch-up campaign) (see Figure 1; Supplementary Figure S1, available online). The CVT reporting timelines were used to inform the timeline of mitigation scenarios at which a switch to a 2-dose vaccination schedule occurred, if the CVT data were to show that a 1-dose vaccine protection was waning. In our base case, we assumed that 1 dose of HPV vaccine provided 30 years of protection (normally distributed at the population level with a standard deviation of 5 years), which would be observed by the trial in the year 2036 by the 30-year data of CVT and would prompt a switch to a 2-dose program. Our base case also assumed programs achieved 80% vaccination coverage in all eligible age groups at program introduction and mitigation using the bivalent HPV vaccine, which provided 100% protection against vaccine-targeted HPV-16 and -18, with no cross-protection against other HPV genotypes. To provide an upper-bound estimate for a possible mitigation strategy, we assumed the 2-dose multi-age cohort mitigation scenarios identified and revaccinated all prior 1-dose vaccine recipients. For women vaccinated after sexual initiation, we assumed the vaccine protected against incident infections but would not affect clearance of a prevalent infection already present at the time of vaccine receipt. In sensitivity analysis, we considered all scenarios under higher (90%) and lower (40%) HPV vaccine coverage assumptions; in addition, the Harvard model explored a 1-dose vaccine protection lasting 25 years (5-year standard deviation) with CVT reporting in the year 2031 and mitigation strategy starting at that time. The scenarios included in the primary and secondary analyses were evaluated against a counterfactual 2-dose or 1-dose HPV vaccination with durable protection (with or without multi-age cohort) that provided 100% lifelong protection against HPV-16 and -18 infections (eg, Figure 1**fourth column**).

### Outcomes

For each scenario, we used model-reported estimates of reductions in age-standardized [WHO 2015 female population (12)] cervical cancer incidence rates per 100 000 women and scaled to the number of cervical cancer cases over 2024-2108 (inclusive) compared with no HPV vaccination program. To provide broad guidance for high-burden settings on the number of cervical cancer cases, we assumed that in the absence of HPV vaccination, 1000 cervical cancer cases would be detected each year through 2108 (excluding underlying population growth). We calculated the average number of cases across the 2 simulations models with uncertainty expressed as the minimum and maximum cases averted from the individual models. We reported the percentage of cumulative cases averted for each scenario by 85 years (2024-2108) compared with the counterfactual 2-dose or 1-dose HPV vaccination with durable protection.

### Model descriptions

The Harvard and HPV-ADVISE models capture HPV natural history and cervical disease, as well as HPV transmission, and have been described in detail previously (3). Both models underwent calibration to reflect sexual behavior, HPV prevalence, and cervical cancer burden from a high-burden setting (age-standardized incidence rate of >50 per 100 000 women), such as Uganda, Tanzania, and Zambia. These models are particularly suited to capture the important dynamics in this analysis as they 1) track intercohort effects under hypothetical 1-dose HPV scenarios with limited duration and 2) have calibrated the models to information on local sexual activity and cancer epidemiology, which can be used to explore the need for and type of mitigation program necessary to avoid most cancers.

The Harvard modeling framework uses a multimodel approach to project the population health impact of alternative HPV vaccination scenarios over time, as previously described (3,13) and in Supplementary Material 2 (available online) for current assumptions. For the current analysis, the multimodeling approach involved the individual-based dynamic model of HPV transmission (Harvard-HPV), and the individual-based model of HPV-induced cervical carcinogenesis (Harvard-CC) from the highest-burden epidemiological profile used in the Harvard model WHO elimination analysis (3). The Harvard-HPV model includes 7 independent HPV genotypes (HPV-16, -18, -31, -33, -45, -52, and -58) and pooled category of 7 other high-risk HPV genotypes. The model projects percent change in HPV incidence by genotype over time associated with each HPV vaccination scenario compared with no vaccination, which are used as inputs into Harvard-CC. Harvard-HPV results are the mean of model projections for 2 good-fitting parameter sets identified through calibration (see Supplementary Material 2, available online). Harvard-CC is an individual-based model that tracks women from age 9 years as they face monthly transitions through cervical cancer–related health states until death (14). We used the Harvard-CC model to project cervical cancer incidence by age over time for each scenario.

HPV-ADVISE LMIC is an individual-based, transmission-dynamic model of HPV infection and disease (see Technical Appendix: http://www.marc-brisson.net/HPVadvise-LMIC.pdf). It models 18 HPV types separately including all nonavalent vaccine types. It reproduces demography, sexual behavior, and HPV transmission (using 4 sexual activity risk groups and sexual mixing by age and risk group), natural history of infection and disease (HPV infection, natural immunity, 3 grades of cervical lesions, and 3 stages of cervical cancer), screening and treatment, and vaccination. HPV-ADVISE was calibrated by identifying the 50 best-fitting parameter sets producing projections that simultaneously fall within prespecified targets (ranges) of the observed sexual behavior and natural history data. HPV-ADVISE simulation results are presented as the mean of all model projections for these 50 parameter sets. A total of 20 replication simulations were performed for each parameter set to reduce stochastic noise.

## Results

### New adopter countries

**Figure 2.**
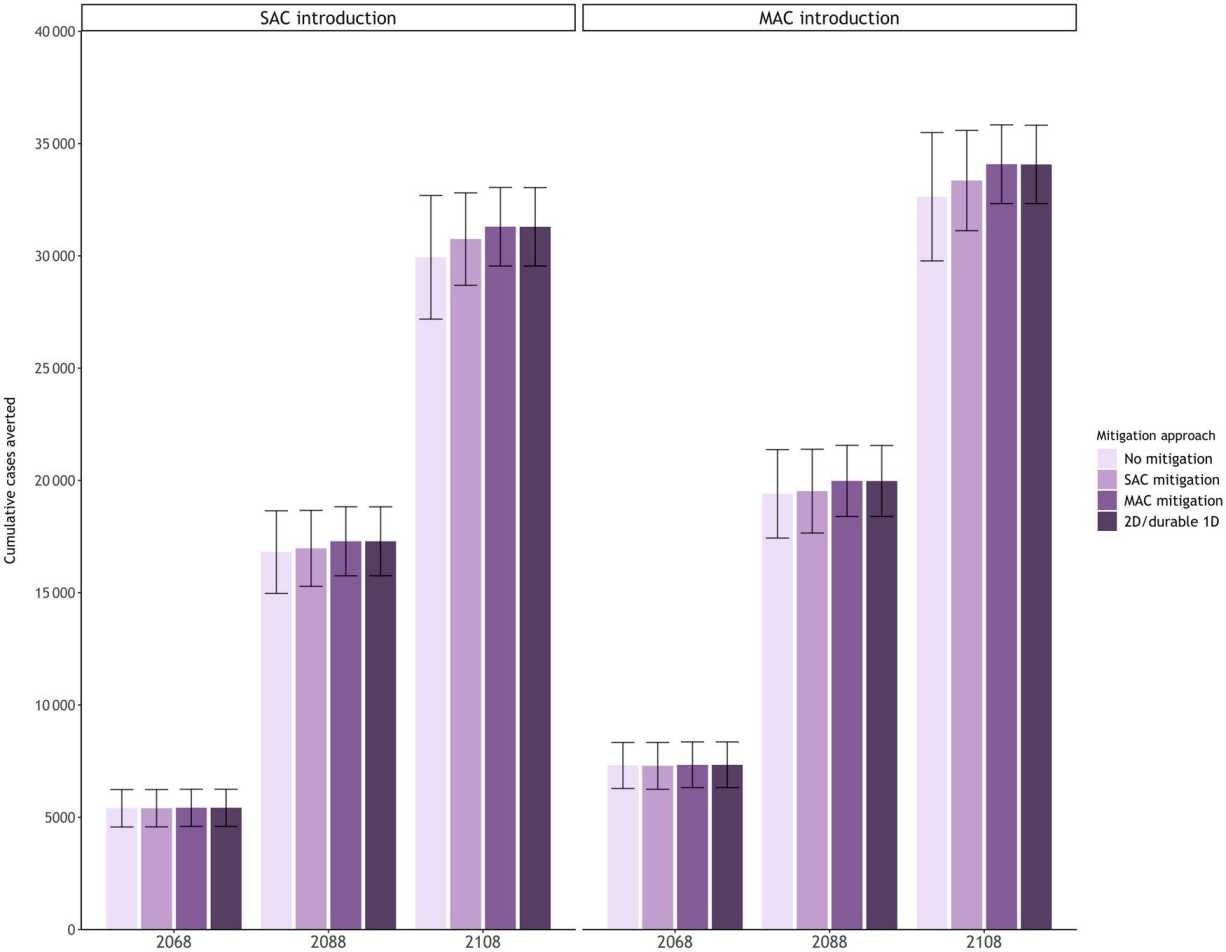
Cumulative cervical cancer cases averted by 2068, 2088, and 2108 for alternative mitigation strategies among new adopter countries by eligible age group(s) at program introduction (single-age cohort for girls aged 9 years [**left panel**] or additionally providing a multi-age cohort for girls aged 10-14 years [**right panel**]) averaged across the Harvard and human papillomavirus (HPV)–ADVISE models (error bars reflect the minimum and maximum across the 2 models). An inferior 1-dose HPV vaccine is assumed to provide an average of 30 years (standard deviation = 5 years) of protection under no mitigation, a 2-dose single-age cohort mitigation, and a 2-dose multi-age cohort mitigation. 1D = 1-dose; 2D = 2-dose; MAC = multi-age cohort; SAC = single-age cohort.

In our primary analysis contextualized for new adopter countries initiating an HPV vaccination program for the first time in 2024, we projected that the cumulative number of cases averted continued to increase over time for all scenarios and was higher for multi-age cohort introduction, while the impact of a hypothetical waning 1-dose vaccine depended on the mitigation approach (Figure 2; Supplementary Figures S2 and S4, available online). For example, if countries introduced a 1-dose schedule assuming an average of 30 years of protection and then switched to a 2-dose schedule in 2036 with a multi-age cohort mitigation approach, both models projected the same level of health benefits as initiating a 2-dose or durable 1-dose program. In contrast, maintaining a 1-dose program under a waning assumption (ie, without switching to a 2-dose schedule) is projected to avert on average of the 2 models 96%-97% (minimum = 94%; maximum = 99%) of cases relative to the 2-dose schedule across the scenarios. However, if countries switched to a 2-dose schedule in the routine program only (ie, single-age cohort mitigation), 98% (minimum = 96%; maximum = 99%) of the cases would be prevented compared with the health benefits of initiating a 2-dose or 1-dose HPV vaccination with durable protection.

### Switcher countries

**Figure 3.**
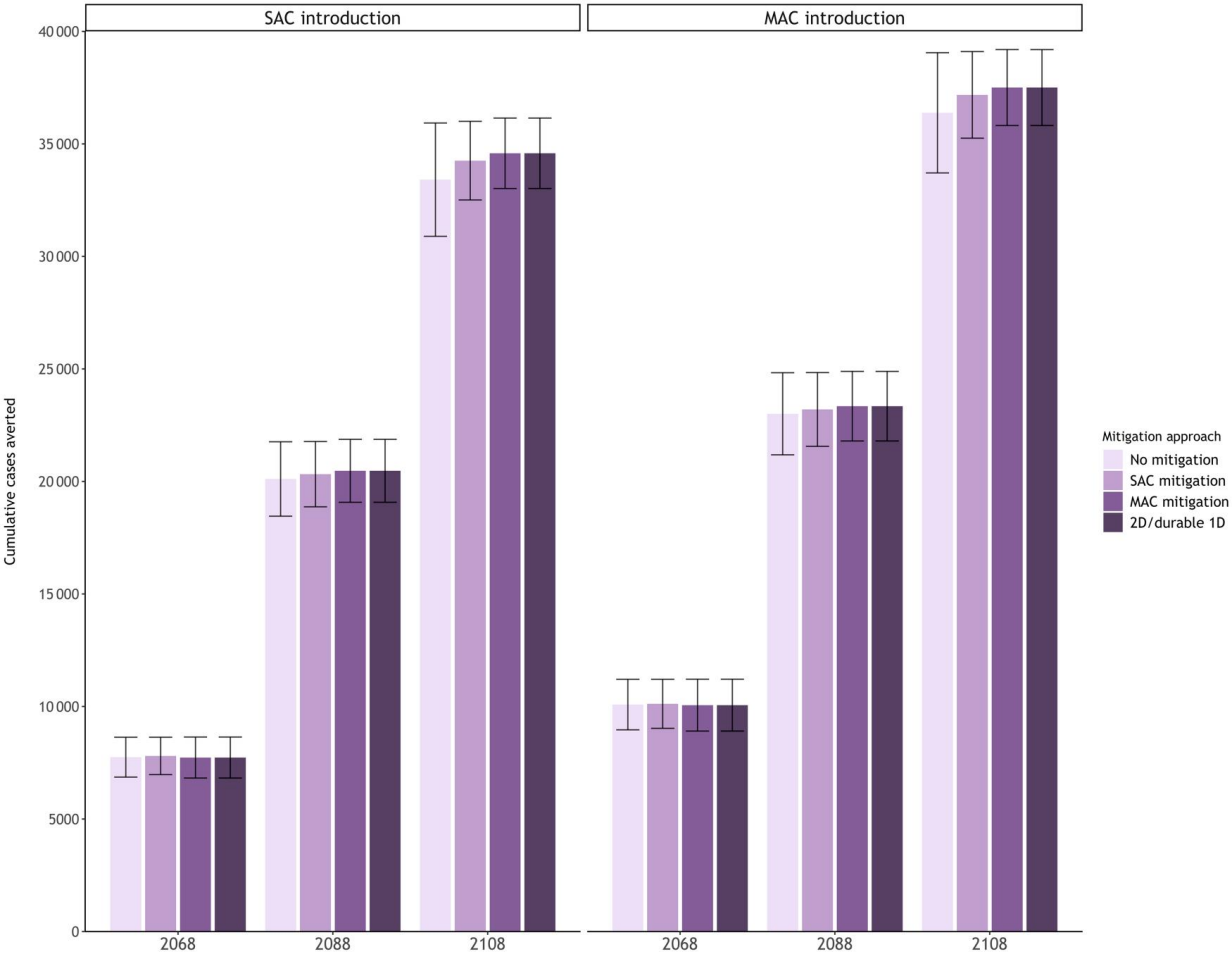
Cumulative cervical cancer cases averted by 2068, 2088, and 2108 for alternative mitigation strategies among switcher countries by eligible age group(s) at program introduction (single-age cohort for girls aged 9 years [**left panel**] or additionally providing a multi-age cohort for girls aged 10-14 years [**right panel**]) averaged across the Harvard and human papillomavirus (HPV)–ADVISE models (error bars reflect the minimum and maximum across the 2 models). An inferior 1-dose HPV vaccine is assumed to provide an average of 30 years (standard deviation = 5 years) of protection under no mitigation, a 2-dose single-age cohort mitigation, and a 2-dose multi-age cohort mitigation. 1D = 1-dose; 2D = 2-dose; MAC = multi-age cohort; SAC = single-age cohort.

Our secondary analysis, contextualized for switcher countries that initiated a single-age cohort 2-dose HPV program in 2019 and switched to a 1-dose schedule in 2024, projected that initiating a single-age cohort HPV program 5 years earlier averted an additional 9%-10% cancer cases by 2108 on average compared with initiating a single-age cohort program in 2024 (Figures 2 and 3; Supplementary Figures S3 and S4, available online). Similar to new adopter countries, we found that if empirical trials reported a waning 1-dose vaccine in 2036, a multi-age cohort mitigation approach averted the same number of cases as maintaining a 2-dose program. The models also projected that the incremental benefits of adding a multi-age cohort mitigation was smaller for switcher countries. For example, a single-age cohort mitigation approach among switcher countries would avert a higher proportion, 99% (minimum = 98%; maximum = 100%) of the 2-dose or durable 1-dose HPV vaccination cases compared with new adopter countries.

### Uncertainty analysis

When we varied the HPV vaccination coverage level among the targeted age cohorts, we found that 40% vaccination coverage prevented 40%-44% (minimum = 33%; maximum = 49%) fewer cases across all scenarios compared with a program achieving 80% coverage (base case), while 90% coverage prevented 6%-7% (minimum = 2%; maximum = 12%) additional cases (Supplementary Table S1, available online). We also found that a multi-age cohort mitigation approach was marginally more impactful for countries achieving 40% vaccination coverage compared with countries achieving 80% coverage (ie, the multi-age cohort mitigation approach had a higher proportion of cumulative cases averted compared with the single-age cohort approach when vaccination coverage was lower [40% vs 80%]), while a single-age cohort mitigation approach averted a similar proportion of cases for countries achieving 90% coverage as for countries achieving 80% coverage (Table 1). Finally, for the Harvard model, when we assumed a 1-dose vaccine provided 25 years of protection (prompting potential mitigation strategy in 2031), a 1-dose program without mitigation prevented fewer cumulative cases compared with a vaccine providing longer protection; however, the incremental benefits of a single-age cohort mitigation approach would avert 98% of the cases for new adopter countries and 99% of the cases for switcher countries with 2-dose or 1-dose HPV vaccination with durable protection compared with the mitigation starting earlier in 2031 (results not shown).

**Table 1.** Proportion of cumulative cases averted by 2108 under a 2-dose single-age cohort mitigation compared with a 2-dose multi-age cohort mitigation for alternative human papillomavirus (HPV) vaccination coverage scenarios (averaged across the Harvard and HPV-ADVISE models)^a^

	Vaccination coverage		
	80%^b^	40%	90%
New adopter countries			
1-dose SAC and SAC mitigation	98%	96%	98%
1-dose MAC^c^ and SAC mitigation	98%	96%	98%
Switcher countries			
1-dose SAC and SAC mitigation	99%	97%	99%
1-dose MAC^c^ and SAC mitigation	99%	98%	99%

a Scenarios vary by whether they were contextualized for 1) countries considering de novo introduction of a 1-dose HPV vaccination program in 2024 (new adopters) or 2) countries with a prior 2-dose HPV program introduction in 2019 followed by a hypothetical switch to a 1-dose schedule 5 years later in 2024 (switchers). One dose is assumed to provide an average of 30 years (standard deviation 5 years) of protection with a switch to the 2-dose mitigation occurring in 2036. Rounded to nearest percent. MAC = multi-age cohort at program introduction ages 10-14 years; SAC = single-age cohort routine vaccination at age 9 years.

b Base case assumption.

c MAC includes routine SAC and 1-year temporary MAC.

## Discussion

Using a comparative modeling approach, we found that an overwhelming majority of health benefits in the hypothetical case of waning 1-dose HPV vaccination are maintained by a switch to 2-dose mitigation strategies that can be introduced when the results of long-term HPV vaccine trials are expected. Furthermore, 2-dose mitigation strategies are not limited to multi-age cohort approaches that require revaccinating women who previously received 1-dose of the HPV vaccine. We found that a 2-dose single-age cohort mitigation approach would achieve 96%-99% of the cases averted as a 2-dose multi-age cohort mitigation approach, depending on baseline programmatic coverage. This analysis also continues to support the health benefits of starting HPV vaccination programs as soon as possible with pre-adolescent multi-age cohorts, which may also reduce the need for a multi-age cohort mitigation in the case of a waning 1-dose HPV vaccination.

Our findings suggest that countries that are hesitant to adopt a 1-dose HPV vaccination because of concerns of potential reduced benefits may have opportunities to leverage the benefits and efficiency gains of a 1-dose vaccination. Consequently, countries could consider capitalizing on a 1-dose schedule without delay and adopt a more simplified programmatic switch of the routine program (single-age cohort mitigation) in the event the long-term trial reports waning protection. Recontacting and reconsenting vaccine recipients under a multi-age cohort mitigation approach, particularly in an LMIC setting, is a challenging health system exercise and would likely require additional research on strategies for re-engagement a population level. Furthermore, a 1-dose multi-age cohort introduction without mitigation averts on average a greater number of cancers than a durable vaccine with a single-age cohort introduction, supporting early 1-dose adoption. It is important to note that a 1-dose vaccine that provides, on average, 30 years of protection would likely show signs of waning in empirical trials prior to the 30-year reporting timeline. For example, if a vaccine provided on average 30 years of protection, approximately 16% of the cohort of women would begin to wane by the 25-year empirical analysis (given a normal distribution and a 5-year standard deviation). Therefore, the timeline for the introduction of hypothetical mitigation strategies in this analysis under a vaccine expected to provide 30 years of protection is likely conservative. In that case, countries might be able to switch prior to the 2036 reporting of the CVT. Under either waning scenario, the majority of HPV exposure occurs before the vaccine begins to wane, which is why the delayed second dose offers small benefits. Our projections are particularly relevant in light of the 2 new prospective trials evaluating the efficacy of a 1-dose nonavalent HPV vaccine that was recently announced by Merck & Co. (Merck Sharp and Dohme [MSD] outside the United States and Canada) (15). Unfortunately, any new trial will not be able to provide insights on 20- or 30-year durability before years 2044-2054. High, quadrivalent 1-dose efficacy in young Kenyan women has already been demonstrated in a high-quality randomized trial (6), and our analysis supports leveraging 1-dose efficiency gains while evidence around durability continues to accrue in the already established long-term empirical trials.

There are several limitations of the simulated scenarios that should be considered. We did not consider loss to follow-up or random revaccination in our multi-age cohort mitigation scenarios; however, we projected the health benefits associated with a single-age cohort mitigation, which can be interpreted as a lower bound under no multi-age cohort catch-up. In contrast, a multi-age cohort mitigation with a random revaccination scenario could be even more effective (10) than the multi-age cohort scenario we considered (ie, perfect multi-age cohort revaccination) in our analysis, because random revaccination would lead to more girls receiving at least 1 dose. As of 2023, LMICs that have implemented an HPV vaccination program have used either the quadrivalent or bivalent vaccines; therefore, we did not consider introduction of a 1-dose nonavalent vaccine in 2019 or in 2024. In addition, we considered the use of only a bivalent vaccine for the hypothetical single-age cohort or multi-age cohort mitigation approaches and thereby may have underestimated the total cancer cases averted if the nonavalent vaccine becomes available; however, the incremental differences are likely marginal as the counterfactual 2-dose approach would likely involve a general programmatic switch from the bivalent to the nonavalent vaccine. In favor of a 1-dose vaccine implementation, there may be added benefits of a 1-dose strategy that involves waiting to provide the mitigation dose when a nonavalent vaccine would be available. Our projections did not include health benefits of noncervical cancer, but we expect the relative impacts of single-age cohort and multi-age cohort mitigation approaches to be broadly comparable. Finally, there are inherent limitations in estimating long-term projections, which have been discussed previously (3,11).

An important strength of our analysis is the use of 2 independently developed models that have been used for a wide range of policy analyses, including the WHO cervical cancer elimination projections (3,16). In most instances, the 2 models’ projections are quite similar; however, the Harvard model consistently finds that all 2-dose mitigation scenarios provide relatively fewer benefits than maintaining a 1-dose vaccine that wanes compared with HPV-ADVISE. Differences between the models likely stem from assumptions of sexual behavior and HPV exposure in women past age 30 years (around when the vaccine wanes). Despite differences across models in the overall impact of HPV vaccination and the relative benefits of the strategies, our findings are strengthened by the agreement of the 2 models on the primary conclusions.

Using a comparative model-based approach, we found that losses in health benefits in the hypothetical case of waning a 1-dose HPV vaccination can be overcome by a switch to 2-dose mitigation strategies that can be introduced when the results of long-term HPV vaccine trials are expected. These findings suggest that countries that are hesitant to adopt a 1-dose HPV vaccination because of concerns of potential reduced benefits may have opportunities to leverage the benefits and efficiency gains of a 1-dose schedule while awaiting the long-term reporting from 1-dose durability studies, such as the CVT.

## Supplementary Material

lgae039_Supplementary_Data

## Data Availability

Model outputs underpinning the analysis are available upon reasonable request.
