# Supplementary material for: Population-level health impact of hypothetical waning 1-dose human papillomavirus vaccination and 2-dose mitigation strategies in a high cervical cancer burden setting
Source: J Natl Cancer Inst Monogr. 2024 Nov 12;2024(67):379–86. doi: 10.1093/jncimonographs/lgae039 (PMC11555273; doi:10.1093/jncimonographs/lgae039)
Supplement: lgae039_Supplementary_Data [file lgae039_supplementary_data.zip › lgae039_Supplementary_Data/Burger2024_1Drecovery_JNCI_R1_supplementary1.docx]

**Supplementary Material 1 to accompany the manuscript:**

“Population-level health impact of hypothetical waning single-dose HPV vaccination and 2-dose mitigation strategies in a high cervical cancer burden setting”

.

**Supplementary Figure S1.** Secondary analysis contextualized for "Switcher" countries, i.e., countries with prior 2-dose (2D) HPV program introduction in 2019, followed by a hypothetical switch to a single-dose (1D) schedule five years later in 2024 (with or without 2-dose mitigation).

**Supplementary Figure S2.** Cumulative cervical cancer cases averted by 2068, 2088, and 2108 for alternative mitigation strategies among "New adopter" countries by eligible age group(s) at program introduction (single-age cohort (SAC) only age 9 years (left panel) or additionally providing a multi-age cohort (MAC) aged 10-14 years (right panel)) for the Harvard (red bars) and HPV-ADVISE (blue bars) models. An inferior single-dose (1D) HPV vaccine is assumed to provide 30-years of protection under no mitigation, a 2-dose (2D) SAC mitigation, and a 2-dose MAC mitigation.


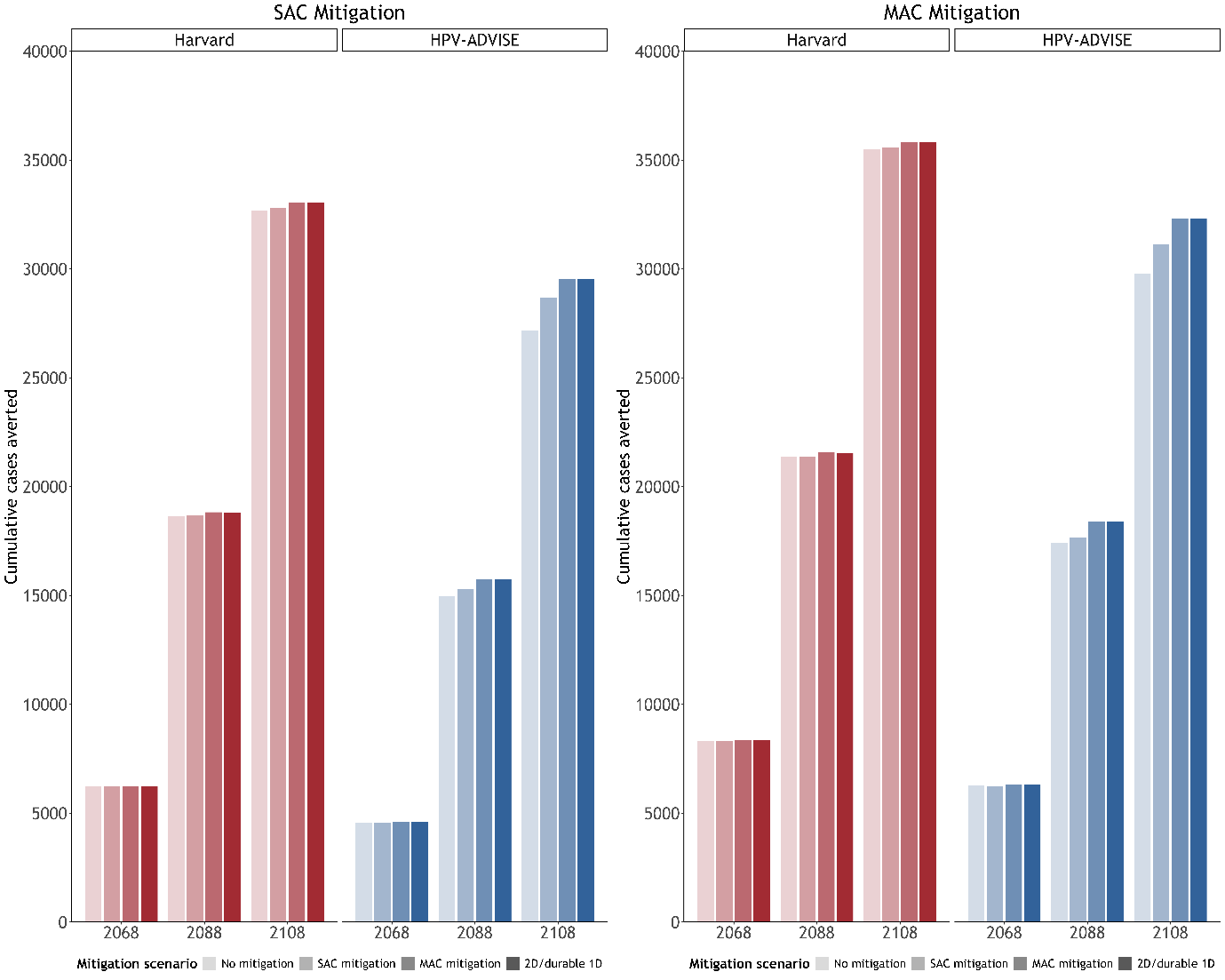


**Supplementary Figure S3.** Cumulative cervical cancer cases averted by 2068, 2088, and 2108 for alternative mitigation strategies among "Switcher" countries by eligible age group(s) at program introduction (single-age cohort (SAC) only age 9 years (left panel) or additionally providing a multi-age cohort (MAC) aged 10-14 years (right panel)) for the Harvard (red bars) and HPV-ADVISE (blue bars) models. An inferior single-dose (1D) HPV vaccine is assumed to provide 30-years of protection under no mitigation, a 2-dose (2D) SAC mitigation, and a 2-dose MAC mitigation.


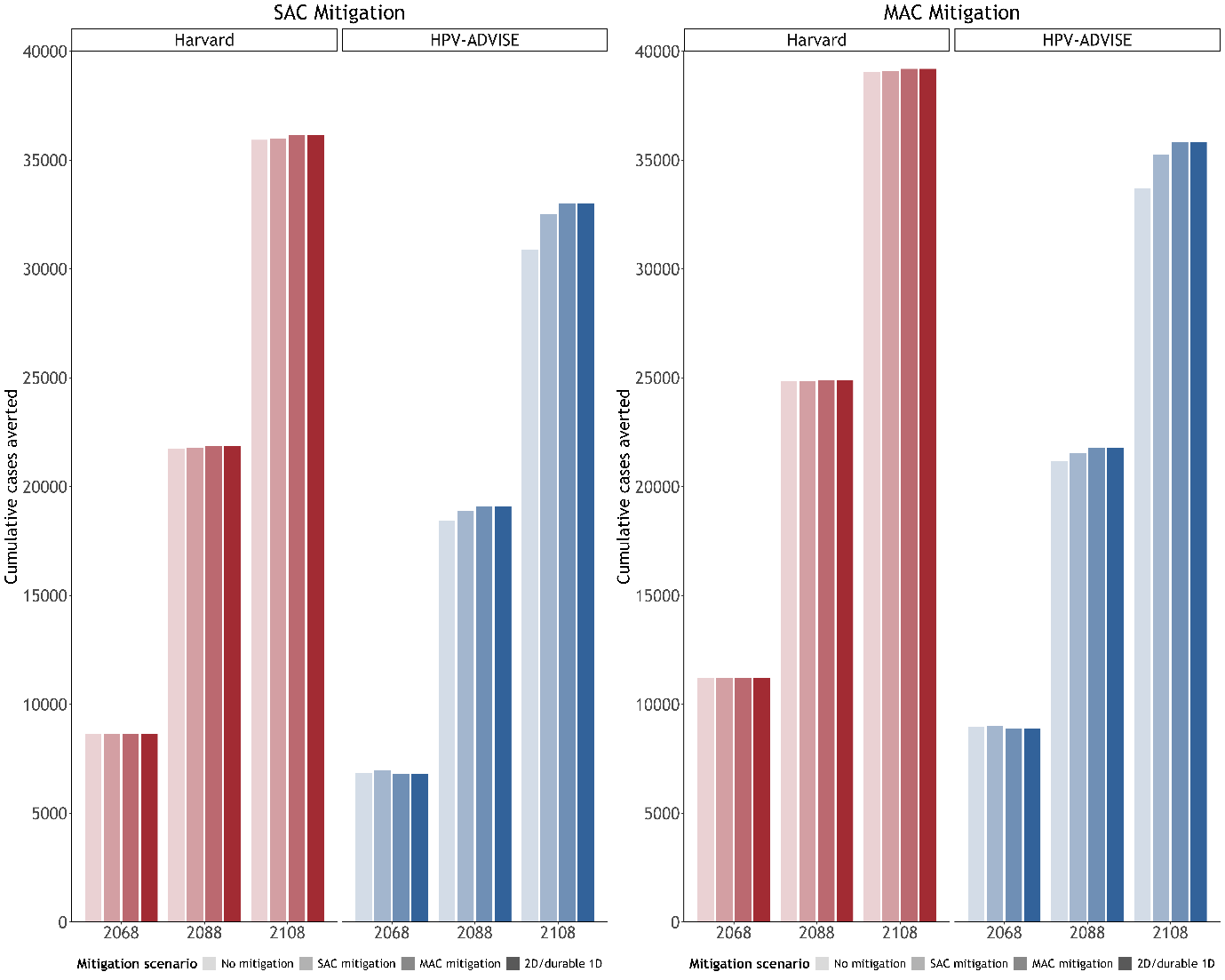


**Supplementary Table S1.** Change in cumulative number of cervical cancer cases by 2108 compared with 80% vaccination coverage under alternative scenarios that varied the eligible age group(s) at program introduction and 2-dose mitigation, i.e., single-age cohort (SAC) only or inclusion of a 1-year multi-age-cohort (MAC) catch-up campaign. An inferior single-dose (1D) HPV vaccine is assumed to provide 30-years of protection under no mitigation, a 2-dose (2D) SAC mitigation, and a 2-dose MAC mitigation.

|  |  | **40% vaccination coverage** | | | **90% vaccination coverage** | | |
| --- | --- | --- | --- | --- | --- | --- | --- |
|  |  | Average | Minimum | Maximum | Average | Minimum | Maximum |
| **"New adopter" countries** | **1D SAC & SAC mit** | -41% | -36% | -48% | 7% | 3% | 11% |
|  | **1D SAC & MAC mit** | -40% | -34% | -47% | 7% | 2% | 11% |
|  | **1D SAC & No mit** | -44% | -40% | -49% | 7% | 3% | 12% |
|  | **2D/durable 1D SAC** | -40% | -34% | -47% | 7% | 2% | 11% |
|  | **1D MAC & SAC mit** | -42% | -36% | -48% | 7% | 2% | 12% |
|  | **1D MAC & MAC mit** | -40% | -33% | -48% | 6% | 2% | 11% |
|  | **1D MAC & No mit** | -44% | -40% | -49% | 7% | 3% | 12% |
|  | **2D/durable 1D MAC** | -40% | -33% | -48% | 6% | 2% | 11% |
| **"Switcher" countries** | **1D SAC & SAC mit** | -41% | -36% | -47% | 6% | 2% | 11% |
|  | **1D SAC & MAC mit** | -40% | -33% | -47% | 7% | 2% | 11% |
|  | **1D SAC & No mit** | -43% | -39% | -48% | 7% | 3% | 11% |
|  | **2D/durable 1D SAC** | -40% | -33% | -47% | 7% | 2% | 11% |
|  | **1D MAC & SAC mit** | -41% | -35% | -48% | 6% | 2% | 11% |
|  | **1D MAC & MAC mit** | -40% | -33% | -48% | 6% | 2% | 11% |
|  | **1D MAC & No mit** | -43% | -48% | -39% | 7% | 2% | 12% |
|  | **2D/durable 1D MAC** | -40% | -33% | -48% | 6% | 2% | 11% |

Note. Rounded to nearest percent

**Supplementary Figure S4.** Model-averaged cancer cases averted for single-age cohort (SAC) compared with multiple-age cohort (MAC) program introductions contextualized for “New adopter” countries, i.e., countries that consider de-novo introduction of a single-dose human papillomavirus (HPV) vaccination program in 2024.

**
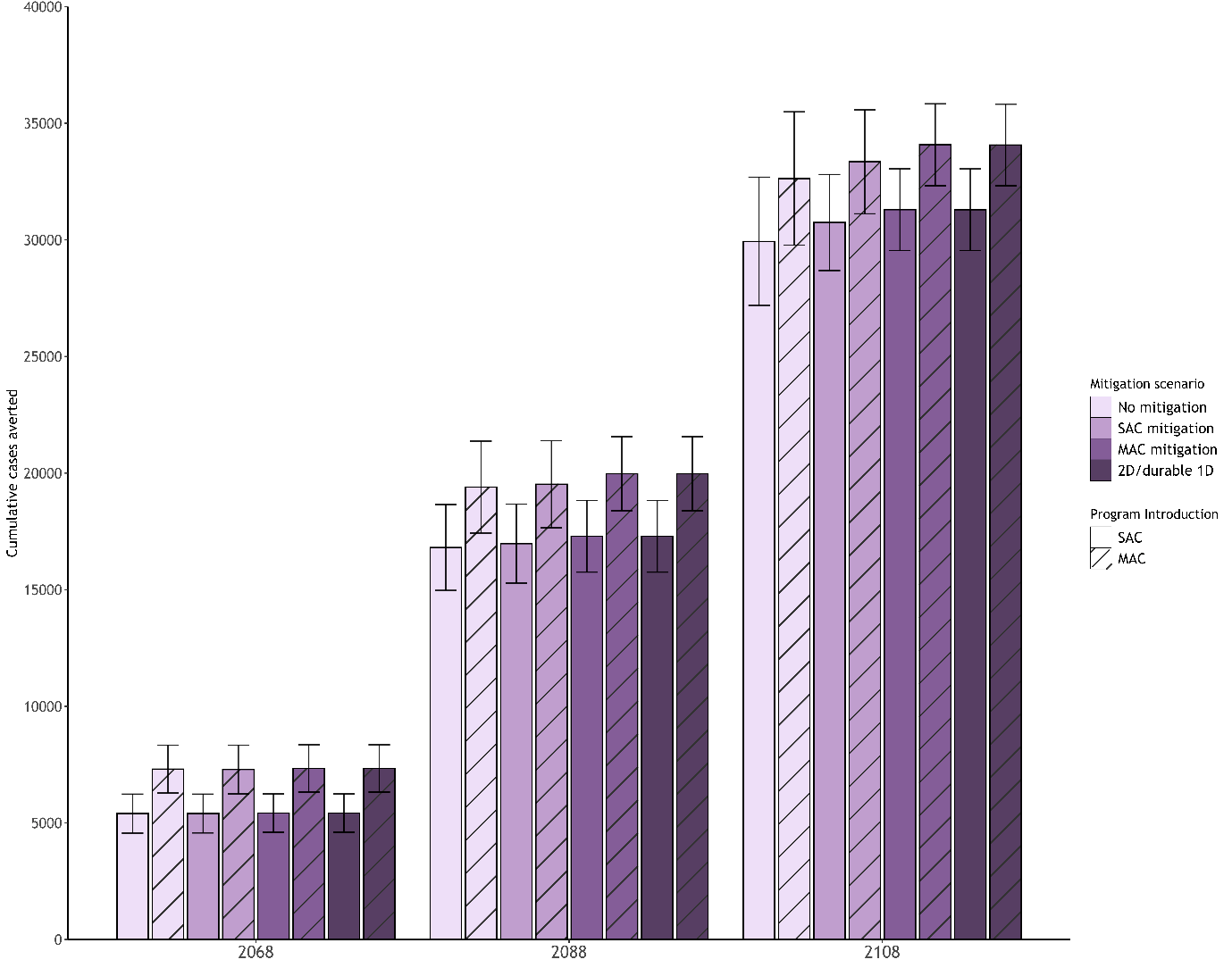
**

**Supplementary Figure S5.** Model-averaged cancer cases averted for single-age cohort (SAC) compared with multiple-age cohort (MAC) program introductions contextualized for “Switcher” countries, i.e., countries with prior 2-dose HPV program introduction in 2019 (based on the introduction timeline for several high-burden countries), followed by a hypothetical switch to a single-dose schedule five years later in 2024 (“Switchers”).

**
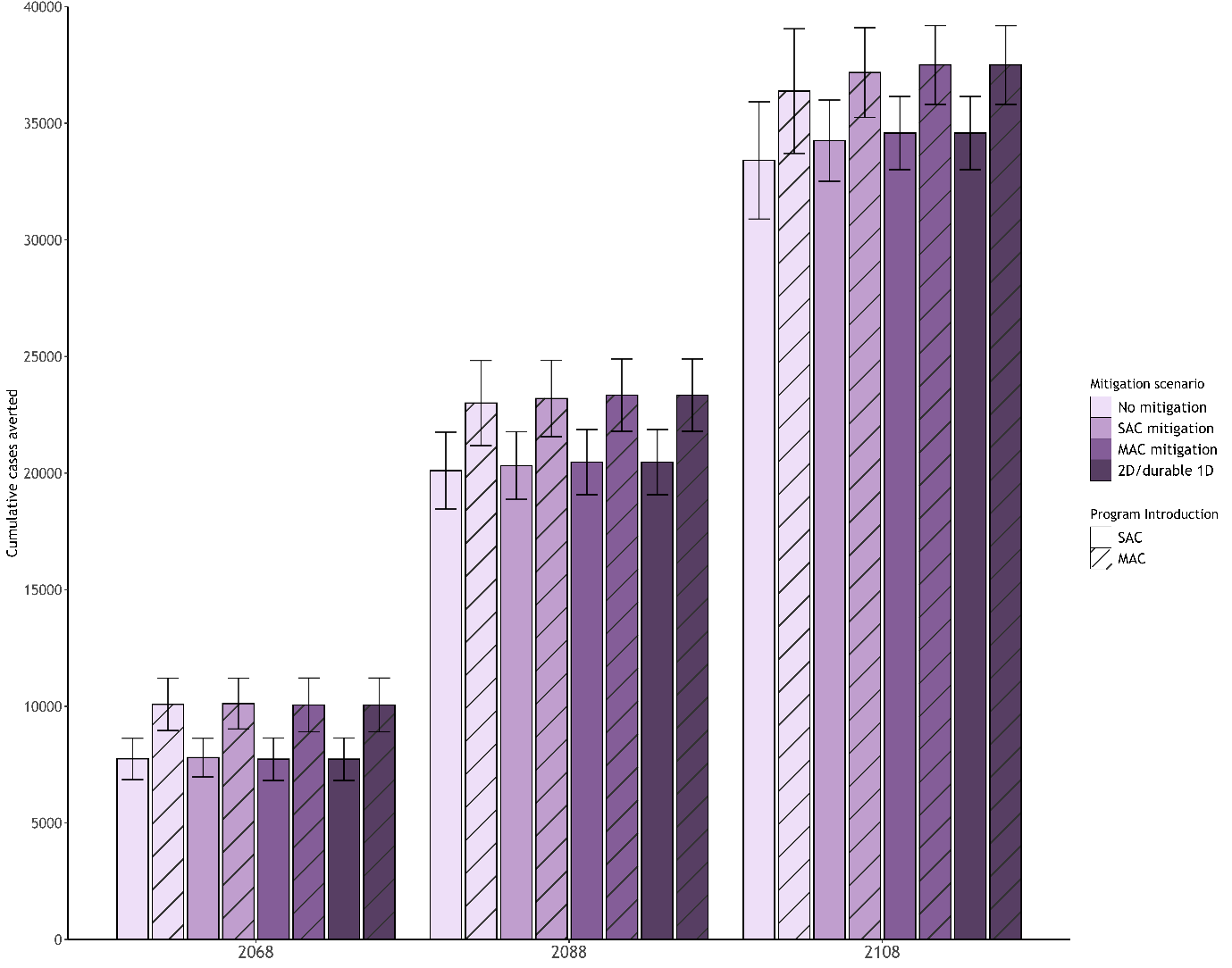
**
