# Supplementary material for: Population-level health impact of hypothetical waning 1-dose human papillomavirus vaccination and 2-dose mitigation strategies in a high cervical cancer burden setting
Source: J Natl Cancer Inst Monogr. 2024 Nov 12;2024(67):379–86. doi: 10.1093/jncimonographs/lgae039 (PMC11555273; doi:10.1093/jncimonographs/lgae039)
Supplement: lgae039_Supplementary_Data [file lgae039_supplementary_data.zip › lgae039_Supplementary_Data/Burger2024_1Drecovery_JNCI_R1_supplementary2_TA.docx]

Technical Appendix for the Harvard dynamic model (Harvard-HPV) for the manuscript:

**“Population-level health impact of hypothetical waning single-dose HPV vaccination and 2-dose mitigation strategies in a high cervical cancer burden setting”**

Contents

[1. Harvard model Overview 2](#_Toc170372261)

[2. Dynamic Model Inputs – Sexual Behavior 4](#_Toc170372262)

[a. Sexual Activity Categories (SAC) 4](#_Toc170372263)

[b. Sexual Partnership Formation 4](#_Toc170372264)

[c. Sexual Partnership Dissolution 7](#_Toc170372265)

[3. Dynamic Model Outputs – Fit to Sexual Behavior 7](#_Toc170372266)

[4. Dynamic Model Inputs – HPV Clearance 9](#_Toc170372267)

[5. Dynamic Model Calibration 10](#_Toc170372268)

[6. References 12](#_Toc170372269)

# Harvard model Overview

As previously described [1], we used a multi-modeling approach involving the linkage of a dynamic transmission model of human papillomavirus (HPV) transmission (“Harvard-HPV”) to an individual-based model of cervical carcinogenesis (“Harvard-CC”) to project the population health and economic consequences for alternative HPV one-dose vaccination scenarios for women over time. Harvard-HPV and Harvard-CC can be used independently, or they can be linked to include direct and indirect benefits from HPV vaccination, and synergies between vaccination and long-term vaccination benefits. Harvard-HPV is an agent-based model that simulates heterosexual HPV transmission and projects the impact of HPV vaccination policy on HPV incidence and prevalence among men and women. Model outputs from Harvard-HPV can inform the complex natural history model of cervical squamous cell carcinoma (Harvard-CC), which simulates individual women from an early age over their lifetime through health states including no HPV infection, HPV infection status, cervical precancer (i.e., cervical intraepithelial neoplasia grade 2 or 3) and cancer [2]. Additional details about Harvard-CC adapted for high-risk countries can be found in Campos, et al. (2015) [2].

The agent-based dynamic model simulates heterosexual partnership acquisition and dissolution, and independent transmission of seven HPV genotypes (HPV-16, -18, -31, -33, -45, -52, -58) (Figure S1). Individuals are stratified by sex, age, and sexual activity category (SAC; four categories: very low (1), low (2), medium (3), high (4)), which govern initial sexual mixing in the population. Each month, individuals in the model cycle through four steps: (1) sexual mixing, (2) HPV infection, (3) HPV clearance and natural immunity, (4) aging, births, and deaths. For each male in the population, the annual number of partnerships (P) is assigned as a function of SAC and age. Partnership assessment occurs at the start of each year of each male’s life, and any new partnerships, if required, are formed randomly during the course of the upcoming year. For males who are missing one or more female partner(s), a new partnership is formed, which continues assuming an age- and SAC-specific probability of dissolution (D) each month. HPV transmission, informed by a sex-specific monthly transmission probabilities, may occur between discordant partners. Sex-specific clearance of an HPV infection allows HPV natural immunity to increase exponentially with each acquisition and clearance of the same HPV genotype. Individuals are eligible to form another partnership, irrespective of ongoing partnerships. Model version 4.5.

For the current analysis, we adapted our dynamic model of HPV transmission [1] to reflect sexual mixing behavior in the high-burden setting of Uganda, where data permitted model fitting (i.e., calibration). Baseline inputs on sexual behavior were derived from the 2016 Ugandan Demographic Health Survey (DHS) and fit to Ugandan-specific HPV prevalence among females [3, 4] and males [5]. For females, calibration targets included an IARC study among women aged 15–24 years and adjusted START-UP prevalence by age and HPV genotype (Odida proportion RLU cutoff 0.5) [3, 4]. For males, calibration targets included a study among uncircumcised men aged 15–49 years in Rakai (HIV+: 30%) [5].

**Figure S1. Harvard dynamic transmission model (Harvard-HPV) schematic for A) Males not missing a female partner and B) Males missing one or more female partners**


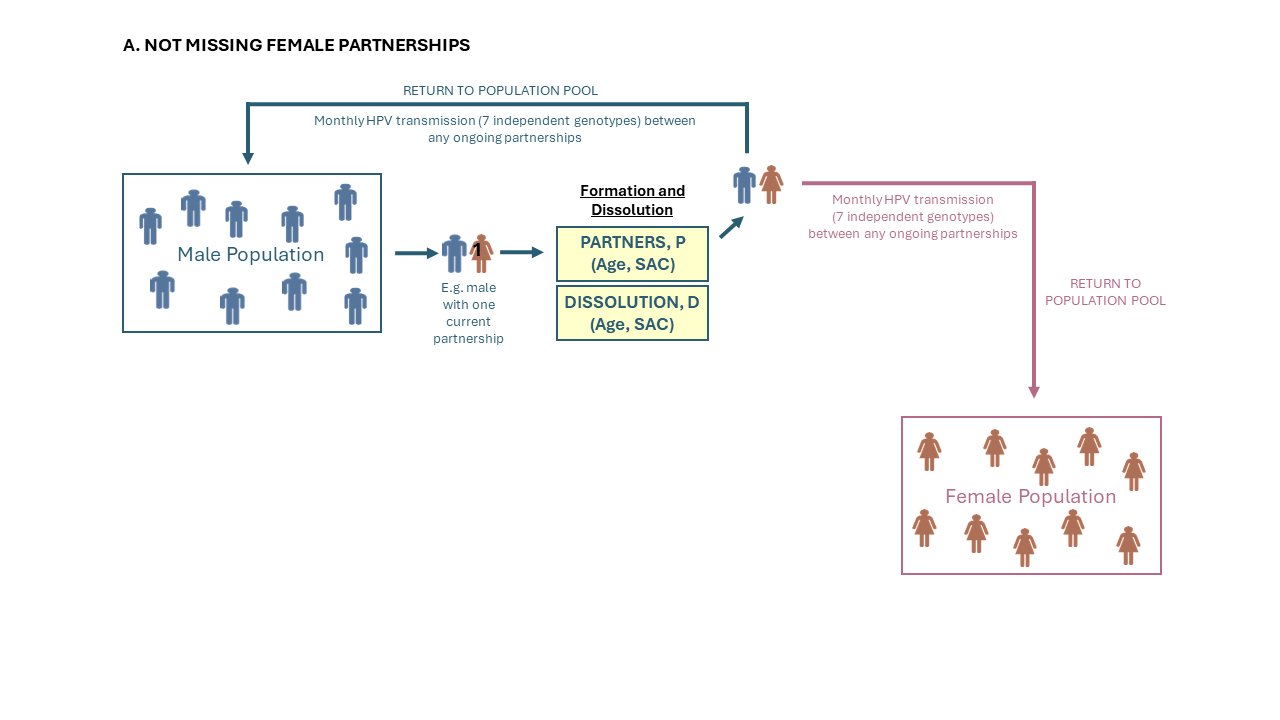


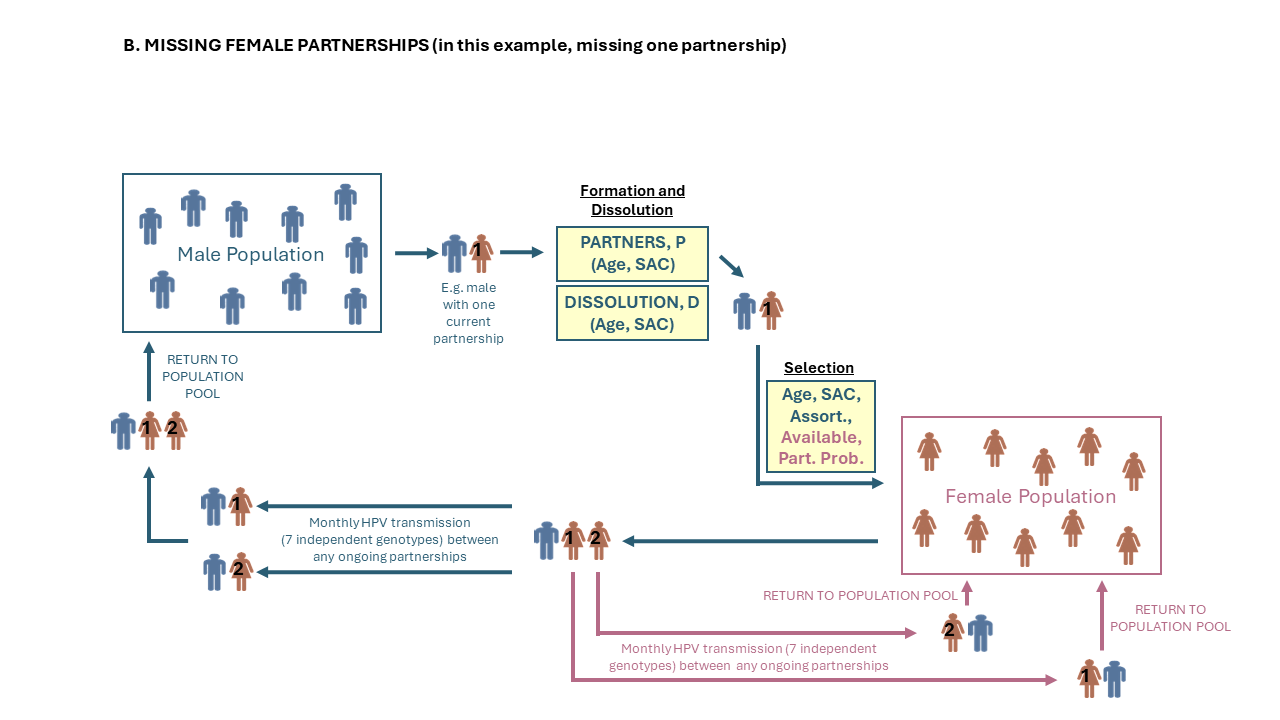


Baseline sexual mixing inputs varied by the number of heterosexual partnerships in the last 12 months by age and four SACs, probability of initiating sexual behavior, the probability of a successful partnership formation, the probability of partnership dissolution by age and SAC, and assortativeness by age (probability of finding partnerships within age bucket, one age-bucket older or one age-bucket younger) and SAC (probability of mixing with partner in the same SAC).

# Dynamic Model Inputs – Sexual Behavior

## Sexual Activity Categories (SAC)

To assess the proportions of the total population engaged in very low (SAC1), low (SAC2), medium (SAC3), and high sexual activity (SAC4), we assessed cumulative lifetime partnerships by sex and age using data from the Ugandan DHS (2016). At each age group, we define sexual activity category by the cumulative number of lifetime partners reported as a percentile of other members of that age group (Table S1). For example, all males at the 90^th^ percentile of lifetime female partners were included in SAC4. At age 20, this would be those with more than 4 lifetime partners, but by age 30 this is those with at least 10 lifetime partners. This results in a consistent portion of the population designated as each SAC at each age group and reflects changing partnership patterns across a lifespan.

**Table S1: Sexual Activity Category (SAC) Distribution by Percentile of Lifetime Partners**

|  | **Female** | **Male** |
| --- | --- | --- |
| SAC 1 | 0–24^th^ | 0–19^th^ |
| SAC 2 | 25–49^th^ | 20–49^th^ |
| SAC 3 | 50–89^th^ | 50–89^th^ |
| SAC 4 | 90–100^th^ | 90–100^th^ |

Note: SAC, Sexual activity category.

## Sexual Partnership Formation

Partnership formation is governed by several factors. Firstly, both males and females have a cumulative probability of initiating sexual behavior that changes by age and SAC categories (Table S2). Onset of sexual behavior is based on a question in the DHS which asks participants to report the age of their first sexual intercourse. However, as initiating sexual behavior is also governed by the availability of eligible partners, the raw numbers from DHS were adjusted to allow for ‘search’ time and correct potential bias in reporting early life sexual behavior.

**Table S2: Cumulative Probability of Initiating Sexual Behavior by Age and SAC** (Uganda DHS 2016)

| Age | SAC1 | SAC2 | SAC3 | SAC4 |
| --- | --- | --- | --- | --- |
| 10 | 0.002 | 0.003 | 0.001 | 0.002 |
| 11 | 0.007 | 0.004 | 0.012 | 0.009 |
| 12 | 0.010 | 0.021 | 0.016 | 0.023 |
| 13 | 0.020 | 0.047 | 0.033 | 0.033 |
| 14 | 0.042 | 0.142 | 0.080 | 0.061 |
| 15 | 0.103 | 0.323 | 0.205 | 0.124 |
| 16 | 0.235 | 0.466 | 0.392 | 0.260 |
| 17 | 0.365 | 0.600 | 0.559 | 0.431 |
| 18 | 0.492 | 0.783 | 0.702 | 0.600 |
| 19 | 0.641 | 0.833 | 0.822 | 0.719 |
| 20 | 0.715 | 0.892 | 0.880 | 0.843 |
| 21 | 0.803 | 0.905 | 0.935 | 0.881 |
| 22 | 0.824 | 0.922 | 0.946 | 0.932 |
| 23 | 0.846 | 0.931 | 0.953 | 0.948 |
| 24 | 0.861 | 0.937 | 0.961 | 0.953 |
| 25 | 0.874 | 0.941 | 0.964 | 0.963 |
| 26 | 0.885 | 0.944 | 0.969 | 0.974 |
| 27 | 0.888 | 0.945 | 0.970 | 0.977 |
| 28 | 0.889 | 0.949 | 0.971 | 0.981 |
| 29 | 0.892 | 0.949 | 0.973 | 0.984 |
| 30 | 1 | 1 | 1 | 1 |

Note: SAC, Sexual activity category.

Based on probability of sexual behavior onset, males in the model will begin seeking female partners, with an upper-bound for number of potential partners determined by sex, age, and SAC category (Table S3) and availability of female partners determined by their potential partnership numbers and sexual behavior onset. Potential number of annual partners was determined using the smoothed estimates of 75^th^ percentile for past-year partners by sex, age, and SAC. As with sexual debut, manual adjustments were made to partnerships at youngest ages to fit the higher estimated lifetime partnerships at youngest ages, particularly for males. Preference for partner’s age and SAC is determined by assortativeness (Table S4), assuming 90% come from within the same SAC category and 22.82% come from within the same 5-year age grouping as the partner-seeking male. An additional 3.62% come from the five-year age grouping one below the partner-seeking male and 46.3% come from the 5-year age group above. Finally, the probability of a successful partnership formation for each desired partnership is governed by “partnership probability” (Table S5) which is applied in each month of “searching” for a partner-seeking male.

**Table S3: Potential Number of Annual Sexual Partners, by Sex, Age and SAC** (Uganda DHS 2016)

|  | Male | | | | Female | | | |
| --- | --- | --- | --- | --- | --- | --- | --- | --- |
| Ages | SAC1 | SAC2 | SAC3 | SAC4 | SAC1 | SAC2 | SAC3 | SAC4 |
| 10–14 | 1 | 1 | 4 | 6 | 1 | 1 | 3 | 4 |
| 15–16 | 1 | 1 | 2 | 3 | 1 | 1 | 2 | 3 |
| 17–19 | 1 | 2 | 2 | 3 | 1 | 1 | 2 | 3 |
| 20–22 | 1 | 2 | 2 | 3 | 1 | 1 | 2 | 4 |
| 23–25 | 1 | 2 | 2 | 3 | 1 | 2 | 2 | 4 |
| 26–31 | 1 | 2 | 2 | 3 | 1 | 2 | 2 | 3 |
| 32+ | 1 | 1 | 2 | 3 | 1 | 2 | 3 | 3 |

Note: Values are rounded to nearest discrete value. SAC, Sexual activity category.

**Table S4: Assortativeness Parameters for Males** (Uganda DHS 2016)

| **Parameter, males** | **Value** |
| --- | --- |
| Probability partner within age bucket | 0.2282 |
| Probability partner one age bucket younger | 0.0362 |
| Probability partner one age bucket older | 0.4630 |
| Probability partner other age | 0.2726 |
| Probability partner same SAC | 0.9000 |
| Probability partner different SAC | 0.1000 |

Note: SAC, Sexual activity category.

**Table S5: Partnership Probability for Males by Age and SAC** (calculated)

| Age | SAC1 | SAC2 | SAC3 | SAC4 |
| --- | --- | --- | --- | --- |
| Age 10–15 | 0.773 | 0.453 | 0.391 | 0.585 |
| 16 | 0.773 | 0.453 | 0.424 | 0.468 |
| 17 | 0.773 | 0.453 | 0.453 | 0.534 |
| 18 | 0.773 | 0.453 | 0.480 | 0.588 |
| 19 | 0.773 | 0.460 | 0.504 | 0.631 |
| 20 | 0.801 | 0.466 | 0.525 | 0.663 |
| 21 | 0.826 | 0.472 | 0.544 | 0.687 |
| 22 | 0.849 | 0.477 | 0.561 | 0.701 |
| 23 | 0.868 | 0.482 | 0.575 | 0.708 |
| 24 | 0.885 | 0.486 | 0.587 | 0.708 |
| 25 | 0.899 | 0.490 | 0.598 | 0.702 |
| 26 | 0.911 | 0.494 | 0.606 | 0.691 |
| 27 | 0.921 | 0.497 | 0.613 | 0.675 |
| 28 | 0.930 | 0.500 | 0.619 | 0.656 |
| 29 | 0.936 | 0.503 | 0.623 | 0.634 |
| 30 | 0.941 | 0.505 | 0.625 | 0.609 |
| 31 | 0.945 | 0.507 | 0.627 | 0.584 |
| 32 | 0.947 | 1.000 | 0.627 | 0.558 |
| 33 | 0.949 | 1.000 | 0.626 | 0.799 |
| 34 | 0.950 | 1.000 | 0.625 | 0.764 |
| 35 | 0.950 | 1.000 | 0.623 | 0.731 |
| 36 | 0.950 | 1.000 | 0.620 | 0.703 |
| 37 | 0.949 | 1.000 | 0.617 | 0.680 |
| 38 | 0.948 | 1.000 | 0.614 | 0.664 |
| 39 | 0.948 | 1.000 | 0.611 | 0.656 |
| 40 | 0.947 | 1.000 | 0.607 | 0.439 |
| 41 | 0.948 | 1.000 | 0.604 | 0.447 |
| 42 | 0.948 | 0.519 | 0.601 | 0.463 |
| 43 | 0.950 | 0.520 | 0.598 | 0.488 |
| 44 | 0.952 | 0.521 | 0.596 | 0.392 |
| 45+ | 0.952 | 0.521 | 0.596 | 0.732 |

Note: SAC, Sexual activity category.

## Sexual Partnership Dissolution

Partnerships end at a monthly separation rate that is determined by the male partner’s age and SAC. Initial separate rates were calculated from data on probability of ending a past-year relationship from the National Survey of Family Growth in the United States, using similarly derived SACs. To calculate the probability of partnership dissolution for Uganda, these probabilities were adjusted according to the differential in lifetime partners in five-year-age-group averages between the United States and Uganda. Final partnership dissolution rates assumed for Uganda are presented in Table S6.

**Table S6: Monthly Probability of Partnership Dissolution, by Age and SAC**

| Age | SAC1 | SAC2 | SAC3 | SAC4 |
| --- | --- | --- | --- | --- |
| Age 10–14 | 0.0018 | 0.0360 | 0.0674 | 0.0777 |
| Age 15–19 | 0.0023 | 0.0450 | 0.0842 | 0.0972 |
| Age 20–24 | 0.0009 | 0.0145 | 0.0260 | 0.0260 |
| Age 25–29 | 0.0008 | 0.0067 | 0.0104 | 0.0156 |
| Age 30–34 | 0.0008 | 0.0042 | 0.0072 | 0.0128 |
| Age 35–39 | 0.0008 | 0.0028 | 0.0053 | 0.0096 |
| Age 40–44 | 0.0009 | 0.0028 | 0.0050 | 0.0081 |
| Age 45–49+ | 0.0011 | 0.0033 | 0.0059 | 0.0095 |

Note: SAC, Sexual activity category. Rounded to nearest ten-thousandth.

# Dynamic Model Outputs – Fit to Sexual Behavior

We compared median and mean lifetime sexual partnerships overall for males and females (Figure S2). Total partnership suggested by the male and female data are incompatible, therefore we have prioritized fitting the male data, but also present fits compared to female data.

**Figure S2: Median (Panel A-B) and Mean (Panels C-D) Lifetime Sexual Partners – Model vs. DHS Data, by Sex**


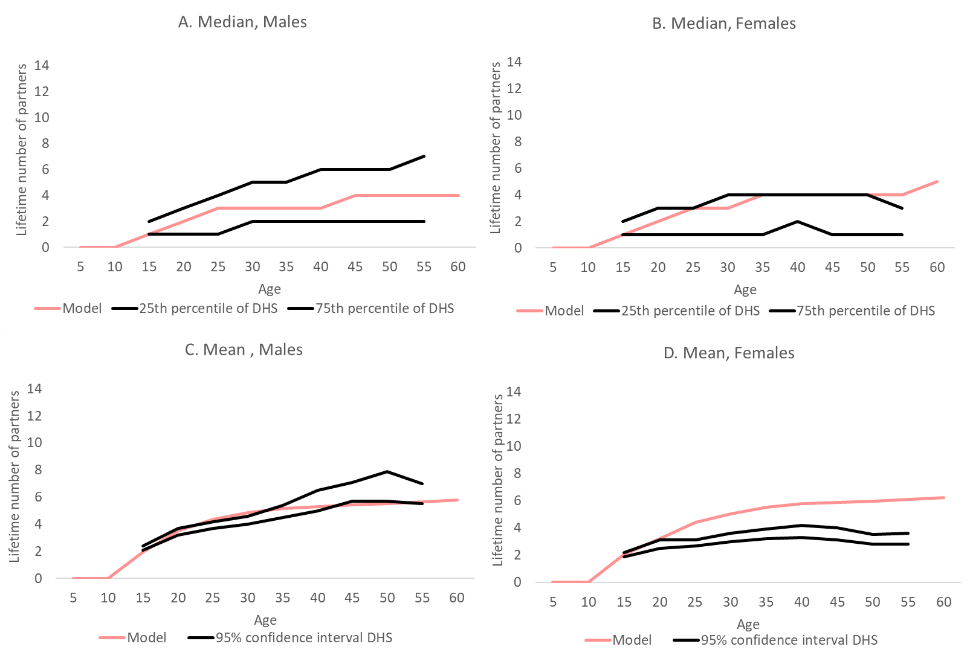


We also evaluated the fit compared to SAC groups (combined Male and Female data) with some inputs adjusted to best fit to mean number of partners by SAC (Figure S3).

**Figure S3: Mean Lifetime Sexual Partners – Model vs. DHS Data, by SAC**


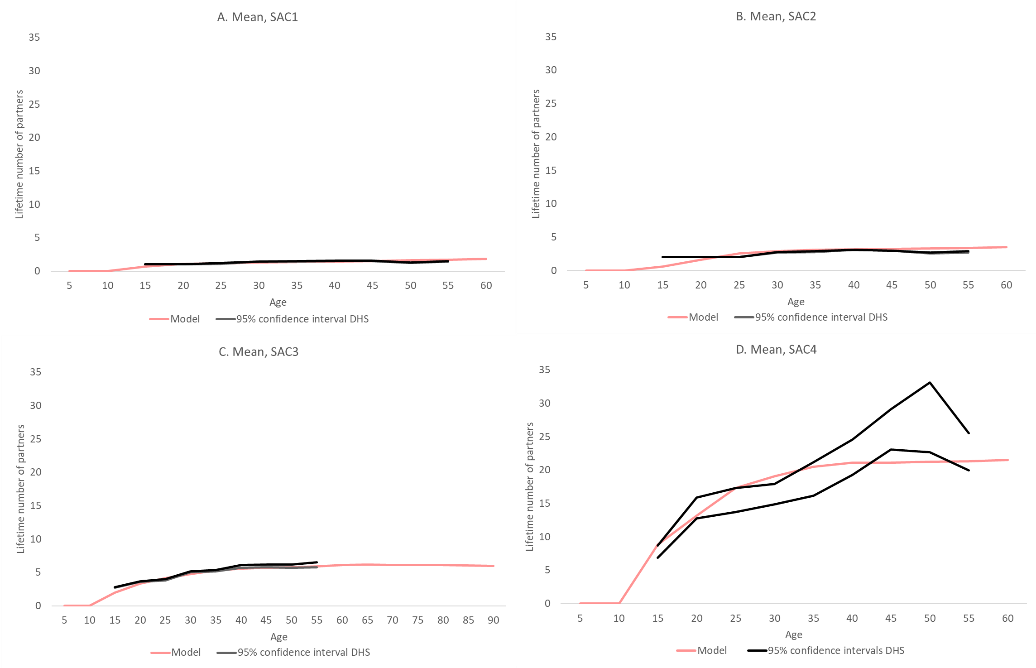


# Dynamic Model Inputs – HPV Clearance

HPV clearance for males and females was estimated using trial data (Table S7). Male HPV infections were assumed to clear 50% faster than female infections, based on Herrero, et al., 2008 [6] and Giuliano, et al., 2011 [7]. We present the calibration outcomes specifically focusing on HPV-16 and HPV-18, as the bivalent HPV vaccine, assuming no cross-protection to non-HPV-16/18 genotypes, was simulated in the analysis.

**Table S7: HPV-16 and HPV-18 clearance**

| **HPV Type, months** | **Male** | **Female** |
| --- | --- | --- |
| **HPV 16** |  |  |
| 1–15 | 0.0628 | 0.0419 |
| 16–27 | 0.0611 | 0.0408 |
| 28–39 | 0.0508 | 0.0339 |
| 40–51 | 0.0478 | 0.0319 |
| 52+ | 0.0298 | 0.0198 |
| **HPV 18** |  |  |
| 1–15 | 0.1100 | 0.0733 |
| 16–27 | 0.0949 | 0.0632 |
| 28–39 | 0.0804 | 0.0536 |
| 40–51 | 0.0309 | 0.0206 |
| 52+ | 0.0309 | 0.0206 |

Note: HPV, human papillomavirus. Rounded to nearest ten-thousandth.

# Dynamic Model Calibration

Baseline inputs, including HPV genotype-specific natural immunity and monthly transmission probabilities were fit (i.e., calibrated) to age- and genotype-specific HPV prevalence [3-5]. Our multi-parameter calibration approach, which has been explained previously, involves a likelihood-based approach to fit to HPV prevalence by uniformly varying the sex- and genotype-specific natural immunity, and uniformly varying the sex- and genotype-specific monthly partnership transmission probability [8]. Following 5,000 model simulations, we identified the 50 best-fitting parameter sets that fit to the calibration targets (Figure S4). Given the uncertainty in the degree of natural immunity of HPV following clearance, we selected the parameter set with the lowest and highest female natural immunity. Analyses were performed using both sets and outcomes averaged (Table S8). Due to high prevalence at young ages and the possibility of obtaining HPV through sexual behaviors beyond insertive vaginal sex (i.e., the question respondents answered in DHS), we assumed a multiplier of 1.5 to increase the monthly transmission (e.g., acts within a month) probability before age 25 years.

**Table S8: Calibrated parameters used in analysis.**

| **Calibration parameter** | **Search range** | **Value parameter set 1** | **Value parameter set 2** |
| --- | --- | --- | --- |
| **Male to female HPV transmission, monthly per partner** |  |  |  |
| HPV-16 | 0.01–female | 0.0507 | 0.0605 |
| HPV-18 | 0.01–female | 0.0872 | 0.1029 |
| **Female to male HPV transmission, monthly per partner** |  |  |  |
| HPV-16 | 0.01–0.10 | 0.0525 | 0.0661 |
| HPV-18 | 0.01–0.10 | 0.1075 | 0.1147 |
| **Natural immunity, males** |  |  |  |
| HPV-16 | 0.00–0.10 | 0.0120 | 0.0092 |
| HPV-18 | 0.00–0.10 | 0.0574 | 0.0209 |
| **Natural immunity, females** |  |  |  |
| HPV-16 | 0.10–0.50 | 0.2565 | 0.4912 |
| HPV-18 | 0.10–0.50 | 0.1287 | 0.2957 |

Note: HPV, human papillomavirus. Rounded to nearest ten-thousandth.

**Figure S4: Harvard-HPV calibration targets (black lines) for females (Panels A**–**B [3, 4]) and males (Panels C**–**D [5]) and model fit (red lines for females and blue dots for males) to age- and genotype-specific HPV prevalence.**


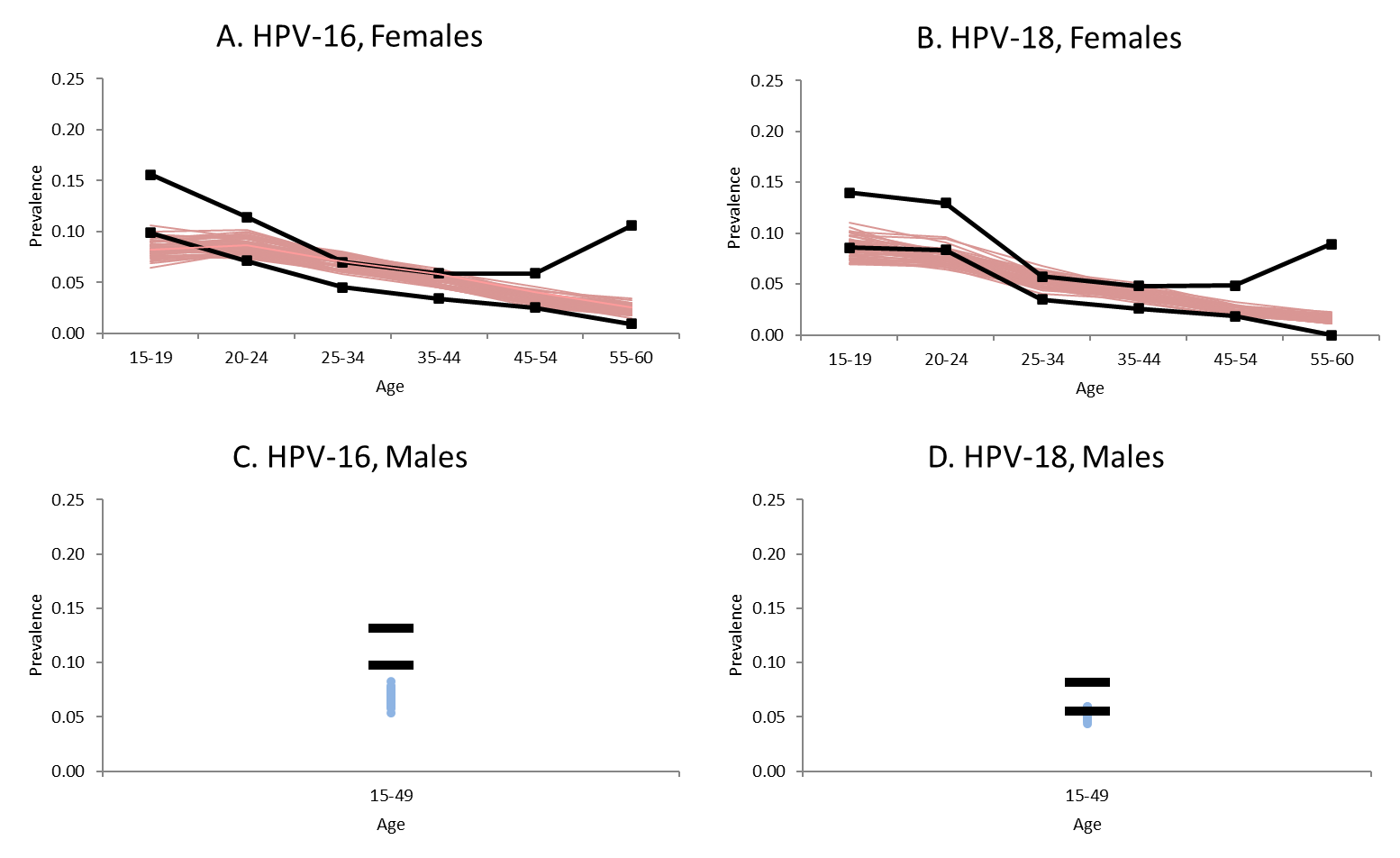


# References

1. Burger EA, Campos NG, Sy S*, et al.* Health and economic benefits of single-dose HPV vaccination in a Gavi-eligible country. Vaccine 2018;36(32 Pt A):4823-4829.

2. Campos NG, Tsu V, Jeronimo J*, et al.* When and how often to screen for cervical cancer in three low- and middle-income countries. A cost-effectiveness analysis. Papillomavirus Research 2015;1:38-58.

3. Jeronimo J, Bansil P, Lim J*, et al.* A multicountry evaluation of careHPV testing, visual inspection with acetic acid, and papanicolaou testing for the detection of cervical cancer. Int J Gynecol Cancer 2014;24(3):576-85.

4. Odida M, Sandin S, Mirembe F*, et al.* HPV types, HIV and invasive cervical carcinoma risk in Kampala, Uganda: a case-control study. Infect Agent Cancer 2011;6(1):8.

5. Tobian AA, Grabowski MK, Kigozi G*, et al.* High-risk human papillomavirus prevalence is associated with HIV infection among heterosexual men in Rakai, Uganda. Sex Transm Infect 2013;89(2):122-7.

6. Herrero R, Hildesheim A, Rodríguez AC*, et al.* Rationale and design of a community-based double-blind randomized clinical trial of an HPV 16 and 18 vaccine in Guanacaste, Costa Rica. Vaccine 2008;26(37):4795-808.

7. Giuliano AR, Lee JH, Fulp W*, et al.* Incidence and clearance of genital human papillomavirus infection in men (HIM): a cohort study. Lancet 2011;377(9769):932-40.

8. Kim JJ, Goldie SJ. Health and economic implications of HPV vaccination in the United States. N Engl J Med 2008;359(8):821-32.
